# Physiological and Pathophysiological Insights of Nav1.4 and Nav1.5 Comparison

**DOI:** 10.3389/fphar.2015.00314

**Published:** 2016-01-14

**Authors:** Gildas Loussouarn, Damien Sternberg, Sophie Nicole, Céline Marionneau, Francoise Le Bouffant, Gilles Toumaniantz, Julien Barc, Olfat A. Malak, Véronique Fressart, Yann Péréon, Isabelle Baró, Flavien Charpentier

**Affiliations:** ^1^Institut National de la Santé et de la Recherche Médicale, UMR 1087, l'Institut du ThoraxNantes, France; ^2^Centre National de la Recherche Scientifique, UMR 6291Nantes, France; ^3^Université de NantesNantes, France; ^4^Institut National de la Santé et de la Recherche Médicale, U1127Paris, France; ^5^Sorbonne Universités, Université Pierre-et-Marie-Curie, UMR S1127Paris, France; ^6^Centre National de la Recherche Scientifique, UMR 7225Paris, France; ^7^Institut du Cerveau et de la Moelle Épinière, ICMParis, France; ^8^Assistance Publique – Hôpitaux de Paris (AP-HP), Centres de Référence des Canalopathies Musculaires et des Maladies Neuro-musculaires Paris-EstParis, France; ^9^Assistance Publique – Hôpitaux de Paris (AP-HP), Hôpital de la Pitié Salpêtrière, Service de Biochimie Métabolique, Unité de Cardiogénétique et MyogénétiqueParis, France; ^10^Centre Hospitalier Universitaire de Nantes, Centre de Référence Maladies Neuromusculaires Nantes-AngersNantes, France; ^11^Atlantic Gene Therapies - Biotherapy Institute for Rare DiseasesNantes, France; ^12^Centre Hospitalier Universitaire de Nantes, l'Institut du ThoraxNantes, France

**Keywords:** Nav1.4, Nav1.5, physiopathology, associated/regulatory proteins, missense mutations

## Abstract

Mutations in Nav1.4 and Nav1.5 α-subunits have been associated with muscular and cardiac channelopathies, respectively. Despite intense research on the structure and function of these channels, a lot of information is still missing to delineate the various physiological and pathophysiological processes underlying their activity at the molecular level. Nav1.4 and Nav1.5 sequences are similar, suggesting structural and functional homologies between the two orthologous channels. This also suggests that any characteristics described for one channel subunit may shed light on the properties of the counterpart channel subunit. In this review article, after a brief clinical description of the muscular and cardiac channelopathies related to Nav1.4 and Nav1.5 mutations, respectively, we compare the knowledge accumulated in different aspects of the expression and function of Nav1.4 and Nav1.5 α-subunits: the regulation of the two encoding genes (*SCN4A* and *SCN5A*), the associated/regulatory proteins and at last, the functional effect of the same missense mutations detected in Nav1.4 and Nav1.5. First, it appears that more is known on Nav1.5 expression and accessory proteins. Because of the high homologies of Nav1.5 binding sites and equivalent Nav1.4 sites, Nav1.5-related results may guide future investigations on Nav1.4. Second, the analysis of the same missense mutations in Nav1.4 and Nav1.5 revealed intriguing similarities regarding their effects on membrane excitability and alteration in channel biophysics. We believe that such comparison may bring new cues to the physiopathology of cardiac and muscular diseases.

Voltage-gated sodium channels (Nav) constitute a family of 10 members in mammals, Nav1.1 to Nav1.9 and Nax, expressed in a large variety of tissues. In excitable cells such as striated myocytes, they initiate action potentials that, in heart as well as in skeletal muscles, trigger, and regulate the contraction. Because of their key role in this function, mutations impacting their activity have tremendous consequences. This review compares the knowledge accumulated in different aspects of the expression and function of Nav1.4 and Nav1.5 α-subunits, and focuses on “homologous” mutations *i.e.*, in the same (aligned) amino acids of the skeletal muscle Nav1.4 and of the cardiac Nav1.5 leading to a large range of muscular and cardiac disorders also called channelopathies.

## Clinical description of the main Nav1.4 and Nav1.5 related pathologies

### Clinical description of Nav1.4 related channelopathies

Nav1.4, which is encoded by the *SCN4A* gene, is the pore-forming subunit of the main sodium channel present in skeletal muscles. Nav1.4 related channelopathies that affect skeletal muscle excitability (Vicart et al., 2005; Jurkat-Rott et al., 2010; Nicole and Fontaine, 2015) are dominant diseases classified in two opposite groups as defined by the prevalent clinical symptoms: muscle stiffness and hypertonia (myotonia) episodes [non dystrophic myotonias (NDM)], and muscle weakness resulting in paralysis episodes (periodic paralyses; PP). It should be noted that similar clinical pattern are also associated with other channelopathies involving chloride channels (NDM) or calcium channels (PP). Table 1 summarizes the main classes of Nav1.4-related skeletal muscle channelopathies. Detailed clinical, electromyographic (Fournier et al., 2004, 2006), genetic and, *in fine*, pathophysiological analyses have led to distinguish several entities among skeletal muscle sodium channelopathies.

**Table 1:** **Main classes of Nav1.4 skeletal muscle channelopathies (Trip et al., 2009; Raja Rayan and Hanna, 2010)**.

	**Clinical manifestations**	**Triggers**	**Paraclinics**	**EMG canonical pattern**	**First intention treatment**	**Most frequently mutated residues**	**References**
PC	Stiffness followed by weakness Paradoxical myotonia	Cold		Myotonia Type I (repeated short effort test)	Mexiletine	T1313 (ID III-IV), R1448 (DIV S4)	McClatchey et al., 1992b; Ptácek et al., 1992; Hayward et al., 1996; Featherstone et al., 1998; Bouhours et al., 2004
SCM	Stiffness at exertion (most often), permanently at rest (*myotonia permanens*), or acetazolamide-responsive myotonia	Exertion Acetazolamide		Myotonia Type III (repeated short effort test)	Mexiletine	G1306 (ID III-IV), G1306A/V: *myotonia fluctuans* G1306E: *myotonia permanens*	Lerche et al., 1993; Rüdel et al., 1993; Ricker et al., 1994; Hayward et al., 1996
HyperPP	Short episodes (minutes)	Fasting	Normal or high potassium level during episodes	Some myotonia Type IV (long effort test)	Acetazolamide	T704 (DIIS5), M1592 (DIV S6)	Ptácek et al., 1991; Rojas et al., 1991; Yang et al., 1994; Iaizzo et al., 1995
HypoPP	Long-lasting episodes (hours, days)	Glucide-rich meals Rest after exercise Prolonged rest	Markedly low potassium levels during episodes	No myotonia Type V (long effort test)	Acetazolamide	R669, R672 (DII S4)	Bulman et al., 1999; Jurkat-Rott et al., 2000; Bendahhou et al., 2001; Sternberg et al., 2001

PC, Paramyotonia Congenita; SCM, Sodium channel Myotonia; Hypo, Normo, Hyper PP, Hypo, Normo, Hyper-kalemic Periodic Paralysis.

#### Nav1.4-related non dystrophic myotonias

Myotonia may occur at the beginning of effort and be alleviated (myotonia, with warm-up effect), or aggravated (paradoxical myotonia, also named paramyotonia) by continuing effort. Those myotonic or paramyotonic symptoms are associated with myotonic discharges analyzed with electromyographic investigations. NDM are opposed to dystrophic myotonias as observed in Steinert (Myotonic Dystrophy type 1, DM1) and PROMM (PROximal Myotonic Myopathy or Myotonic Dystrophy type 2, DM2) diseases. Among NDM, at least two entities differ clinically and electromyographically (Trip et al., 2009; Raja Rayan and Hanna, 2010).

- Paramyotonia congenita (PC) consists of cold-induced stiffness often associated with some weakness of face and extremities muscles, and paradoxical myotonia; it is associated with a progressive decrease of compound muscle action potential (CMAP) amplitude during repetitive short efforts test at EMG (pattern I according to Fournier, Fournier et al., 2004).
- Sodium channel myotonias (SCM) regroup the remaining dominant sodium channel-related myotonias that are not significantly cold-sensitive or paradoxical, and do not exhibit any change of CMAP amplitude during repetitive short efforts test at EMG (pattern III according to Fournier); this SCM entity was initially termed “potassium-aggravated myotonia” as potassium load triggers myotonia in some cases. This group was further subdivided into three types: *myotonia permanens, myotonia fluctuans*, and acetazolamide-responsive myotonia. While this classification is not used in clinics, it has some relevance: *myotonia permanens* designates myotonia that is present permanently, even at rest; *myotonia fluctuans* designates myotonia that appears and disappears at some moment, with no systematic concomitance with exertion, a peculiar circumstance being exercise-induced delayed-onset myotonia, that occurs some time after exertion has stopped; acetazolamide-responsive myotonia is a treatment-related designation, that underlines the fact that some SCM are treatable by acetazolamide.

#### Nav1.4-related periodic paralysis

Among PP, two distinct entities are recognized (Raja Rayan and Hanna, 2010): hypokalemic periodic paralysis (HypoPP) is characterized by a marked hypokalemia concomitant with paralysis episodes, and, on the opposite, hyperkalemic periodic paralysis (HyperPP) is associated with a tendency to high blood potassium levels during the paralysis episodes. From the electromyographic point of view, both are characterized by a marked decrease of CMAP amplitude after a 5 min-long effort (long effort test, also referred to as McManis test).

Overlap, borderline or mixed syndromes between PP and NDM or between their subtypes have been reported (McClatchey et al., 1992a; Sugiura et al., 2003; Webb and Cannon, 2008; Yoshinaga et al., 2012). The age at onset is usually in early to late childhood. Neonatal symptoms are not classically reported in the most frequent Nav1.4 channelopathies, but dominant *de novo* mutations are reported in moderate to severe neonatal clinical presentations such as severe neonatal episodic laryngospasm (SNEL) (Lion-Francois et al., 2010). In a general way, respiratory symptoms are not common in PP and NDM, however a small number of patients are exposed to laryngeal or diaphragmatic weakness or myotonia that may be symptomatic.

The minimal prevalence of skeletal muscle Nav1.4 channelopathies has been recently estimated to be 0.4:100,000 in England (Horga et al., 2013) and 1.4:100,000 in France. Mutations in Nav1.4 are mostly missense or rarely in-frame deletions or insertions, usually with a dominant effect. However exceptional recessive homozygosity (Arnold et al., 2015) and a possible recessive compound heterozygosity (Tsujino et al., 2003) have been reported in congenital myasthenic syndromes. A small number of canonical mutations account for a significant percentage of cases (Table 1), e.g., T1313M and R1448C/H for PC, T704M for HyperPP, V445M (Rosenfeld et al., 1997), V1293I (Koch et al., 1995), and G1306A/V/E for SCM, mutations of domains II and III S4 arginines (IIS4 and IIIS4) at position 669 (R>H), 672 (R>H/G/C/S), 1132 (R>Q) (Carle et al., 2006), 1135 (R>H) for HypoPP (Matthews et al., 2009). Mutations at IIS4 arginine 675 (R>Q/G/W) result in a special type of PP with both features of HyperPP and HypoPP (Vicart et al., 2004). However, beside those frequent canonical mutations, more than 70 different missense mutations at more than 55 different positions in different domains of the protein have been reported in the literature as causative mutations for Nav1.4 channelopathies. The penetrance of Nav1.4 dominant mutations is variable for each mutation: it is high for HyperPP (T704M), PC (T1313M/A and R1448C/H) and SCM (V445M and V1293I) mutations, and lower, with cases of gender-related non-penetrance in pedigrees, for some other mutations such as HypoPP mutations at position 669 or 672 (Ke et al., 2013).

### Clinical description of Nav1.5 related channelopathies

Nav1.5, which is encoded by the *SCN5A* gene, is the pore-forming subunit of the main cardiac sodium channel. Nav1.5 related channelopathies affecting cardiac excitability are dominant diseases that, similarly to Nav1.4 in the skeletal muscles, impact cardiac excitability through loss of function or gain of function effects on Nav1.5 activity. Table 2 summarizes the Nav1.5 related channelopathies that are discussed in this review, which only considers pathologies provoked by mutations in the same, i.e., aligned amino acids in Nav1.4 and Nav1.5 (cf. Part Comparison of Missense Mutations. Are there (dys-)Functional Homologies between Nav1.4 and Nav1.5?): the Brugada syndrome (BrS), the long QT syndrome (LQTS), and arrhythmic dilated cardiomyopathy. The latter includes a novel form of cardiac arrhythmia characterized by multifocal ectopic Purkinje-related premature contractions (MEPPCs), associated or not with atrial fibrillation and dilated cardiomyopathy. Consequently, Table 2 is not an exhaustive list of Nav1.5 related channelopathies.

**Table 2:** **Nav1.5 cardiac channelopathies**.

	**Clinical manifestations**	**Triggers**	**Paraclinics**	**ECG canonical pattern**	**First intention treatment**	**References**
Brugada syndrome (BrS)	Ventricular fibrillation or aborted sudden cardiac death, syncope, nocturnal agonal respiration, palpitations	Rest or sleep, febrile state, vagotonic conditions		ST-segment elevation on right precordial leads (V1 and V2)	Implantable cardioverter-defibrillator (ICD)	Brugada and Brugada, 1992; Antzelevitch et al., 2005
Type 3 Long QT syndrome (LQTS3)	Polymorphic ventricular tachycardia (torsades de pointes), ventricular fibrillation, syncopes, sudden death	Rest or sleep, bradycardia, hypokaliemia, drugs prolonging QT interval		Prolonged QT interval	β-blockers (with or w/o mexiletine)	Wang et al., 1995; Amin et al., 2013; Giudicessi and Ackerman, 2013
Arrhythmic Dilated Cardiomyopathy	Systolic dysfunction, left ventricular enlargement or dilatation. Multiple arrhythmias (text)	For MEPPC: rest (exercise suppresses PVCs)			For MEPPC: Quinidine Amiodarone	McNair et al., 2011; Laurent et al., 2012; Mann et al., 2012; Nair et al., 2012; Beckermann et al., 2014

This list is not exhaustive, but corresponds to pathologies caused by Nav1.5 mutations that are homologous to mutations in Nav1.4 (cf. **Tables 4**, **5**).

#### The brugada syndrome

The BrS is a primary electrical disorder that is characterized by a specific ECG pattern consisting of ST-segment elevation followed by a negative T-wave in the right precordial leads (Brugada and Brugada, 1992), indicating abnormal electrical activity in the upper part of the right ventricle (right ventricular outflow tract). This ECG pattern is associated with an increased risk of sudden cardiac death (SCD) resulting from polymorphic ventricular tachyarrhythmias or ventricular fibrillation. The incidence of BrS in the general population is currently estimated at 1:2000 (Antzelevitch et al., 2005). This syndrome is 8–10 times more prevalent in males than in females and typically manifests during adulthood, with a mean age of SCD of 41 ± 15 years (Antzelevitch et al., 2005). BrS was first described as a monogenic disease, with autosomal dominant transmission. Although more than 20 genes have been proposed as causally related to BrS, mutations in these genes explain less than 30% of the cases (Crotti et al., 2012; Nielsen et al., 2013; Antzelevitch and Yan, 2015; Veerman et al., 2015). Around 25% of BrS patients possess a mutation in *SCN5A*. So far, ≈300 mutations in *SCN5A* have been reported as related to BrS (http://www.ncbi.nlm.nih.gov/clinvar). These mutations lead to a loss of Nav1.5 function and reduce Na^+^ current (I_Na_). Besides BrS, loss-of-function mutations in *SCN5A* also cause isolated cardiac conduction disease and sinus node dysfunction (Remme et al., 2008). ECG signs of conduction defects are also a common feature of BrS. The other genes identified so far are coding for proteins that are involved in generating or regulating the sodium current (Antzelevitch and Yan, 2015), the L-type calcium current (Antzelevitch et al., 2007; Burashnikov et al., 2010; Béziau et al., 2014) or the transient outward potassium current (Delpón et al., 2008; Giudicessi et al., 2011).

If BrS was first described as a monogenic autosomal dominant disease, there is accumulating evidence suggesting that it follows a more complex genetic model. Concerning *SCN5A*, segregation studies performed in large affected pedigrees demonstrate that mutations in this gene are characterized by a low penetrance (47%). In some instances, a single *SCN5A* mutation can lead to different cardiac arrhythmia phenotypes in the same family or even in a single patient (Kyndt et al., 2001; Probst et al., 2009). Moreover, in some pedigrees, the absence of the familial *SCN5A* mutation is observed in some affected family members, suggesting other origins for the disease (Probst et al., 2009). Recently, a genome-wide association study in a large cohort of BrS patients has provided the proof of concept that common genetic variants outside the *SCN5A* gene, e.g., *SCN10A* and *HEY2* loci in the reported study, may have a large effect on the development of the disease (Bezzina et al., 2013). Altogether, these data suggest that the BrS most probably involves combined contribution of different gene variants of variable impact.

#### The long QT syndrome

Congenital LQTS is defined by several criteria including a prolongation of the QT interval corrected for heart rate, i.e., QTc, to values above 440 ms in males and 460 ms in females, due to prolonged ventricular action potentials. LQTS patients are predisposed to ventricular polymorphic tachyarrhythmias (*torsades de pointes*) that may lead to syncope, seizure or SCD (Amin et al., 2013). The most common form of LQTS (also called Romano-Ward syndrome) is an autosomic dominant disease. Its incidence in the population worldwide is about 1:2000 (Schwartz et al., 2009). To date, genetic defects in 15 different genes have been found in 70% of the LQTS patients (Amin et al., 2013; Giudicessi and Ackerman, 2013). Similar to BrS, the disease penetrance is most often incomplete and highly variable, ranging from 25 to 100% (Priori et al., 1999; Viadero et al., 2011). This suggests that additional genetic and non-genetic factors may modify the clinical manifestations of a given LQTS-causing mutation. In recent years, numerous studies have shown that genetic variants play an important modulatory role in establishing the disease severity (Amin et al., 2013). Among non-genetic factors, hypokalemia, or treatment with drugs inhibiting K_V_11.1 (hERG) channels as side effect are well known to favor arrhythmic events. Sex is also a well-known modifier of QT interval duration in LQTS. Post-adolescence and pre-menopause women have a lower repolarization reserve than men and are therefore more prone to QT interval prolongation and cardiac events. This is partially explained by the effects of sex hormones on cardiac ion channel expression and function (Tanabe et al., 1999; Zicha et al., 2003; Bai et al., 2005; Gaborit et al., 2010). The most common types of LQTS are LQTS1 (30–35% of patients; Ackerman et al., 2011), LQTS2 (25–40%), and LQTS3 (5–10%), due to defects in *KCNQ1* (K_V_7.1 channel), *KCNH2* (K_V_11.1), and *SCN5A* (Nav1.5) genes, respectively. Approximately 80% of all LQTS causal mutations are found in these three genes. Clinically, LQTS3 is characterized by unusually increased duration of the ST segment with a late appearance of the T wave (Moss, 2002). It is often more lethal, although less frequent, than LQTS1 and LQTS2 (Priori et al., 2003). Bradycardia and pauses occurring at rest or more particularly during sleep are often at the origin of the arrhythmias, although fatal tachycardia-induced arrhythmias have also been reported for a third of the patients. Most of the *SCN5A* mutations that were reported to be related to LQTS3 (≈200; http://www.ncbi.nlm.nih.gov/clinvar) alter the fast inactivation process of the channel, leading to persistent inward sodium current causing prolonged membrane depolarizations (Wang et al., 1995; George, 2005).

#### Arrhythmic dilated cardiomyopathy

Dilated cardiomyopathy (DCM) is characterized by systolic dysfunction and, in most patients, left ventricular enlargement or dilatation. It has been associated with the mutations of more than 30 genes, including *SCN5A* (McNair et al., 2011; Hershberger et al., 2013). Sixteen *SCN5A* mutations are linked to familial or sporadic cases with DCM with various types of arrhythmias, for example, sinus node dysfunction, conduction delay, and atrial and/or ventricular tachy-arrhythmias (Amin, 2014). Among arrhythmic DCM, the MEPPC syndrome is a recently-described autosomal dominant form of cardiac arrhythmia (Laurent et al., 2012). It is characterized by frequent premature ventricular contractions (PVCs) originating from various ectopic foci along the fascicular-Purkinje system occasionally associated with dilated cardiomyopathy, non-sustained ventricular tachycardias (NSVTs), and sudden death. A similar phenotype was first reported in 2003 by Bezzina and collaborators in a newborn boy and his diseased sister, both genotyped with Nav1.5 W156X and R225W mutations (Bezzina et al., 2003). Both parents and an elder sibling, each one carrier of one or the other mutation, were asymptomatic. For the sister, arrhythmias being the cause of the DCM is unlikely because persistent arrhythmias were only present for a short period. Two other mutations in Nav1.5 (R222Q and R225P) have been linked to this MEPPC syndrome in several families (Laurent et al., 2012; Mann et al., 2012; Nair et al., 2012; Beckermann et al., 2014). In these families, dilated cardiomyopathy, when present, was suggested as a consequence of severe primary electrical dysfunctions.

### Phenotypic and genotypic overlap between cardiac and skeletal muscle sodium channelopathies?

A recently published study shows that patients carrying (or not) *SCN4A* causative mutations, present with mixed phenotype (BrS and myotonic features) (Bissay et al., 2015). Although *SCN4A* transcripts are present in human ventricles (Péréon et al., 2003), it is difficult to understand how the gain of function *SCN4A* mutations can be compared to the loss of function of *SCN5A* mutations classically associated with Brugada, as discussed in the study of Bissay and collaborators. Another study on a unique family described four patients carrying a *SCN4A* mutation and presenting with PC (Péréon et al., 2003), two of them having slightly prolonged QTc interval. Both PC and LQTS3 are associated with a gain of function of Nav1.4 and Nav1.5, respectively. In this case, it is tempting to hypothesize that the mutant Nav1.4 channels present in the heart are responsible for the QT prolongation. Identifying more families with such overlap phenotypes would help to confirm the potential mutual influence of both channels on the pathogenesis of cardiac and muscular diseases.

## Channel molecular bases and gene expression

Voltage-gated sodium channels consist of an α-subunit, constituting the pore, and accessory β-subunits controlling the expression and activity of the pore-forming subunit. Nav1.4, the most frequent Nav α-subunit expressed in the skeletal muscle is a glycosylated transmembrane protein of 1836 amino acids and has an apparent molecular weight of approximately 260 kDa (George et al., 1992a,b). Nav1.5, the most frequent Nav cardiac α-subunit is 2015–2016 amino acid long, depending on the splice variants, and has a similar apparent molecular weight (Gellens et al., 1992; Makielski et al., 2003; Balasuriya et al., 2012).

The *SCN4A* gene which encodes Nav1.4 is composed of 24 exons, all containing coding sequence. No alternative splicing events have been reported in the literature. Nav1.5 is encoded by the *SCN5A* gene, composed of 28 exons, among which exons 2–28 contain the coding sequence. Exon 1 and part of exon 2 encode the 5′ untranslated region (UTR) while exon 28 contains the 3′-UTR (Wang et al., 1996). Intron 2 of *SCN4A* and intron 3 of *SCN5A* are AT-AC type I introns. Intron 21 of *SCN4A* and intron 25 of *SCN5A* are AT-AC type II introns (Wu and Krainer, 1999). All other introns are canonical GT-AG introns. Unlike Nav1.4, mRNA variants of Nav1.5 are detected in the heart of mammals, resulting from alternative splicing. In human and murine hearts, 3′-UTRs present two different splicing variants, generating short or long poly-adenine tails (Shang and Dudley, 2005). In addition, three rare variants were identified only in human, corresponding to alternative splicing of exon 28A by exons 28B–28D coding for truncated and non-functional forms of Nav1.5 (Shang et al., 2007). To date, only the mechanisms of this splice site are understood. They involve interactions with two splicing factors, the RBM25 and LUC7F3 proteins (Gao et al., 2011; Gao and Dudley, 2013). Four and three splice variants, which differ from the canonical non-coding sequence, were described for the 5′UTR of human and mouse *SCN5A* mRNAs, respectively. These transcripts originate from the alternative splicing encompassing exons 1 (designated 1A, 1B, 1C, and 1D) and 2, and are preferentially expressed in the heart as compared with other tissues. Also, a neonatal isoform containing a neonatal exon 6A of 31 nucleotides has been reported. This form presents a difference of seven amino acids in the S3–S4 loop of domain I, in comparison with exon 6 of the adult form (Rook et al., 2012). Ventricular myocardial analysis displayed abnormal splicing of *SCN5A* exon 6, characterized by over-expression of this neonatal isoform, in one patient who present DCM with conduction system disease (Wahbi et al., 2013). These findings suggest a potential implication of mis-splicing of *SCN5A* in the cardiac defect observed in this patient.

Two distinct sodium currents and channels were historically described in skeletal muscle depending upon the developmental and innervation status of the myofiber. SkM1, the TTX-sensitive sodium channel expressed in innervated adult myofibers, corresponds to Nav1.4 and is the main skeletal muscle sodium channel (Trimmer et al., 1989; Kallen et al., 1990). SkM2, the TTX resistant sodium channel expressed in immature and denervated myofibers, corresponds to Nav1.5. In rodents, *SCN4A* expression increases just after birth concomitantly with the decrease of *SCN5A* gene expression (Stocksley et al., 2005). *SCN4A* expression is not sensitive to myofiber denervation by contrast to *SCN5A* gene expression, which was found to be upregulated in response to denervation (Awad et al., 2001).

The *SCN4A* promoter contains distinct positive-acting promoter E-box and negative-acting repressor E-box that cooperate to yield specific gene expression in differentiated skeletal myofibers (Kraner et al., 1998, 1999). It is suggested that the muscle specificity of *SCN4A* expression result from the binding of two basic helix-loop-helix transcription factors (bHLH) of the muscle-specific MyoD family, myogenin and MRF4 for initiation and maintenance, respectively, to the positive-acting promoter E-box located upstream the translation initiation site. NFI would be another major regulator of *SCN4A* gene expression acting in concert with bHLH factors, especially MRF4 (Hebert et al., 2007). The density of Nav1.4 is around 20 times higher at the neuromuscular junction (NMJ), in part as a result of local mRNA accumulation (Stocksley et al., 2005). Although the promoter element responsible for the transcriptional regulation of subsynaptic genes in response to neuronal factors at the NMJ is the N-box (TTCCGG) (Méjat et al., 2003), no N-box is present within the promoter of *SCN4A*, suggesting the involvement of other regulatory elements.

Similarly to alternative splicing, more is known concerning the regulation of the *SCN5A* promoter, compared with *SCN4A*. After the identification of a first promoter region for human *SCN5A* which includes multiple positive and negative cis-acting elements extending into intron 1 (Yang et al., 2004), two other promoter regions for murine *SCN5A* (designated P2 and P3) containing two distinct cardiac-specific enhancer regions were identified and functionally characterized (Shang and Dudley, 2005). In human and rat, the segment immediately upstream of the major transcription start site contains three GC boxes that could serve as binding sites for the Sp1 transcription factor, which are homologous to the CACC boxes recognized in promoters of muscle restricted genes, and an E-box binding site for bHLH factors (Yang et al., 2004). The human sequence also includes an additional C-rich motif which is recognized as a major regulator of expression in myocytes. Further, Yang and collaborators have characterized a binding site for GATA in intron 1, which is also known as a key regulator of gene expression in the heart. Surprisingly, variants in *SCN10A* (encoding Nav1.8 of which expression is extremely low in heart and undetectable in atrioventricular bundle) are associated with alterations of cardiac conduction parameters and BrS (van den Boogaard et al., 2014). Van den Boogaard and collaborators have shown that the *SCN10A* variants act more likely through an alteration *SCN5A* gene expression level. They have demonstrated that a cis-regulatory element located in *SCN10A* gene -which is immediately located next to *SCN5A*- was able to interact with both *SCN5A* and *SCN10A* promoters. Furthermore, they described, using healthy human heart samples, a direct correlation between the *SCN5A* (but not *SCN10A*) expression and the presence of the rs6801957 risk-associated SNP in the *SCN10A* intronic enhancer. Together, their data provided a genomic mechanism explaining how a common genetic variant at *SCN10A* locus influences cardiac physiology and predispose to Brs.

## Associated/regulatory proteins

**Figure 1:**
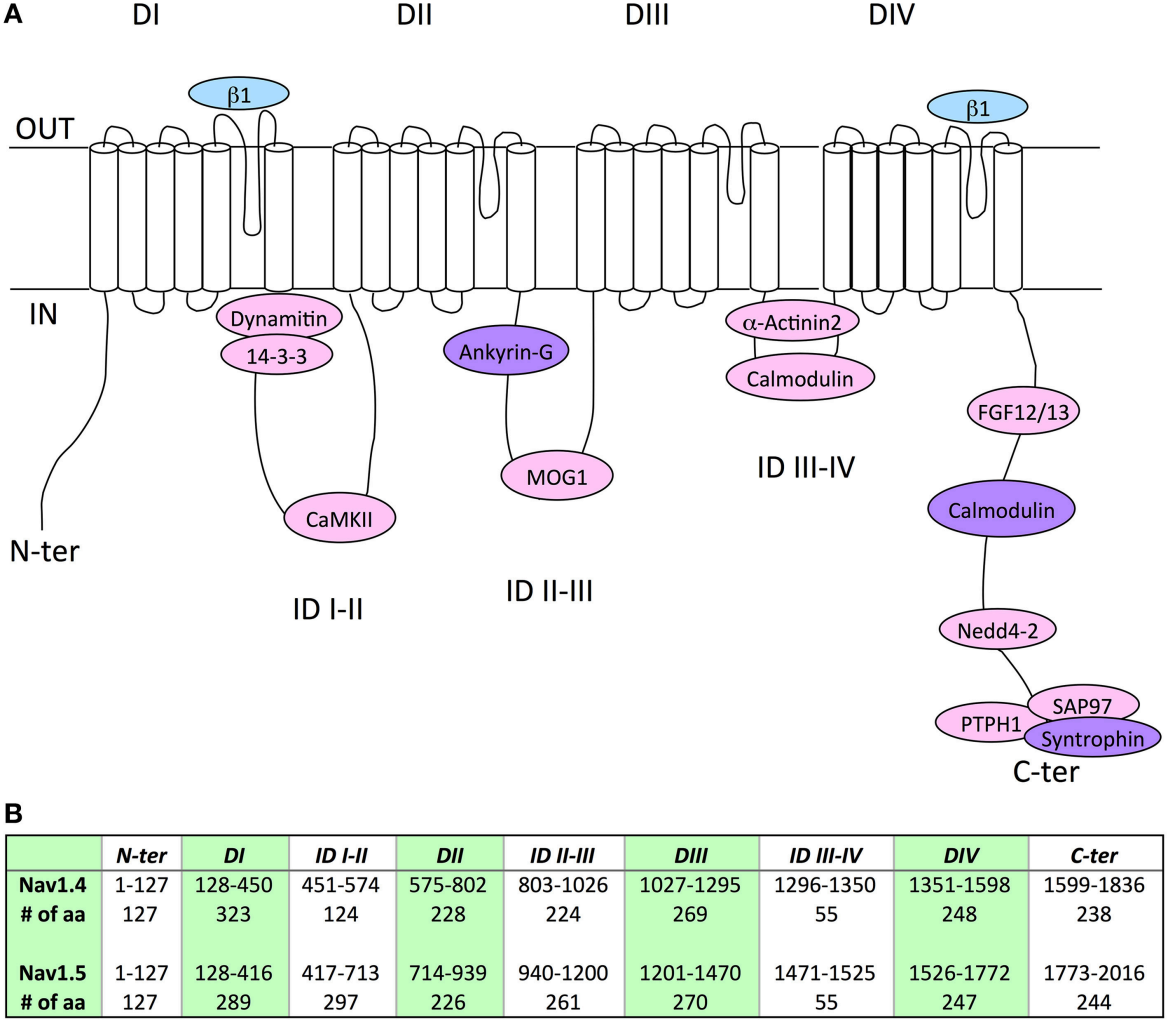
**(A)** Schematic representation of the localization of the binding sites of associated/regulatory proteins identified for Nav1.4 (blue), Nav1.5 (pink), or both (purple). Nav1.4 interaction with ankyrin is only suggested by chimeric constructs, it remains to be studied with full length proteins (Lemaillet et al., 2003). DI to DIV, domains I to IV; ID, intracellular interdomains; N-ter and C-ter, N-and C-terminus ends. **(B)** Table presenting the amino-acid (aa) numbering and length of specific regions and domains in Nav1.4 and Nav1.5 proteins. It is noticeable that Nav1.4 IDI-II total amino-acid length is shorter than Nav1.5 IDI-II.

Although expression of Nav1.5 or Nav1.4 α-subunits alone results in the generation of functional channels in heterologous expression systems, it is now quite clear that the regulation of gating and/or expression of the Nav subunits substantially relies on a variety of other accessory/regulatory proteins (Abriel, 2010; Rook et al., 2012). Interestingly, the alignment of Nav1.5 and Nav1.4 amino acid sequences could facilitate the identification of novel associated/regulatory proteins of the counterpart channel subunit. In addition, this direct sequence comparison has contributed, as for Navβ1 (Makita et al., 1996), and will certainly continue to contribute to localizing the structural determinants involved in the channel regulation. In this respect, Table 3 and Figure 1 recapitulate the Nav1.5 or Nav1.4 amino acid sequences previously identified to mediate interaction with associated/regulatory proteins, and indicates the corresponding sequences in the other Nav α-subunit. Whereas, a number of Nav1.5 interacting proteins, with their binding sites in the channel subunit, have been described in the literature (see references in Table 3), very little is known for Nav1.4 (Figure 1). Nevertheless, it is interesting to note that the amino acid sequence similarity obtained for some binding sites is high, suggesting the possibility that both channel subunits share the same associated/regulatory proteins. This is the case for example of calmodulin, which associates with the very well conserved (100% sequence similarity) IQ-motif on both Nav1.5 and Nav1.4 C-terminal domains (Tan et al., 2002; Young and Caldwell, 2005). Most of the proteins shown to interact with Nav1.5 on a site conserved in Nav1.4 are ubiquitously expressed (dynamitin, 14-3-3, CaMKII, MOG-1, calmodulin, FGF, PTPH1/PTPN3, SAP97), suggesting that an interaction with Nav1.4 may take place in the skeletal muscle cells (Marfatia et al., 2001; Blair et al., 2006). When not ubiquitously expressed, proteins known to interact with Nav1.5 are also expressed in skeletal muscle (α actinin2) that argue for a possible interaction with Nav1.4 (Foley and Young, 2014). Conversely, weaker sequence similarity may suggest different affinities, sites or absence of interaction/regulation. This is the case for example of Navβ1 for which the region within D1/S5-S6 that confers regulation of Nav1.4 in *Xenopus* oocytes (Makita et al., 1996) is not very well conserved in Nav1.5 (63.1% sequence similarity) which is also regulated by Navβ1, suggesting that the structural determinants of the interaction of Nav1.5 or Nav1.4 with Navβ1 are different. Finally, it is striking to note the complete absence of the PY-motif from the C-terminus of Nav1.4. This suggests that the regulation of Nav1.4 channel internalization and/or degradation is achieved through different mechanisms as compared to Nav1.5 for which cell surface expression is regulated through the ubiquitin-proteasome pathway (van Bemmelen et al., 2004; Rougier et al., 2005). These mechanisms remain to be identified.

**Table 3:** **Comparison of Nav1.5 and Nav1.4 channel associated/regulatory proteins and corresponding binding sites**.

**Region**	**Nav1.4/1.5 interacting proteins**	**Nav1.5**				**Nav1.4**				**% aa sequence similarity**
		**Binding sites**	**Mutations**	**Pathologies**	**References**	**Binding sites**	**Mutations**	**Pathol-ogies**	**References**	
DI S5–S6 loop	Navβ1	Equivalent sequence: (278–388) HKC—GKI (111 aa)	R282H, V294M, G319S R282H G292S K317N L325R G351V T353I D356N R367C, M369K R367H R376H R376H L276Q, H278D, R282C, V300I, L315P, K317- T320N, E346X, G351D R367C, R367L, M369K W374G, G386R, G386E	BrS BrS BrS BrS BrS BrS BrS BrS BrS BrS BrS BrS BrS BrS BrS BrS BrS	Priori et al., 2002 Itoh et al., 2005a Niimura et al., 2004 Yi et al., 2003 Keller et al., 2005 Vatta et al., 2002 Pfahnl et al., 2007 Makiyama et al., 2005 Smits et al., 2002 Takehara et al., 2004 Frustaci et al., 2005 Rossenbacker et al., 2004 Kapplinger et al., 2010 Kapplinger et al., 2010 Kapplinger et al., 2010 Kapplinger et al., 2010 Kapplinger et al., 2010	**(278–422)** **QKC–GKT** **(145 aa)** (Makita et al., 1996				63.1%
ID I-II	Dynamitin	**(417–444)** **EEQ—KKE** **(28 aa)** Chatin et al., 2014	E428K, H445D, L461V E439K E446K E462K	AF BrS DCM LQT3	Darbar et al., 2008 Kapplinger et al., 2010 McNair et al., 2011 Tester et al., 2005	Equivalent sequence: (451–478) AEQ–KKH (28 aa)				85.7%
	14-3-3	**(417–467)** **EEQ—PLA** **(51 aa)** Allouis et al., 2006				Equivalent sequence: (451–482) AEQ—EAD (32aa)				66.7%
	CaMKII	**(417–711)** **EEQ–GVK** **(295 aa)** Ashpole et al., 2012				Equivalent sequence: (451–572) AEQ—IIH (122 aa)				87.1% in ID first 31 aa (418–449:452–484) 80.0% in ID last 60 aa (655–714:517–575)
ID II-III	Ankyrin-G	**(1047–1055)** **VPIAVAESD** **(9 aa)** Mohler et al., 2004	S941N R971C A997S T1069M R1023H	LQT3 LQT3 LQT3 LQT3 BrS	Schwartz et al., 2000 Tester et al., 2005 Ackerman et al., 2001 Tester et al., 2005 Frustaci et al., 2005	**(925–933)** **VPIASEESD** **(9 aa)** Lemaillet et al., 2003	S804N	SCM	Fournier et al., 2006	77.8%
	MOG1	**(940–1200)** **SSF—CYH** **(261 aa)** Wu et al., 2008	E1053K, R965C D1055G, R965H, A997T S1079Y, A1113V, S1140T D1114N A1180V R1193Q	BrS BrS BrS LQT3 DCM LQT3	Priori et al., 2002 Kapplinger et al., 2010 Kapplinger et al., 2010 Splawski et al., 2000 Ge et al., 2008 Wang et al., 2004	Equivalent sequence: (803–1026) SSF—CFK (224 aa)				50.2%
ID III-IV	α-Actinin-2	**(1471–1523)** **DNF—IFD** **(53 aa)** Ziane et al., 2010	G1481E F1486L Y1494N M1498T L1501V	LQT3 LQT3 BrS LQT3 LQT3	Tester et al., 2005 Wang et al., 2007 Tian et al., 2007 Napolitano et al., 2005 Splawski et al., 2000	Equivalent sequence: (1296–1348) DNF—VYD (53 aa)	N1297K G1306E G1306E G1306E	SNDM SCM SNEL PC	Gay et al., 2008 Mitrovic et al., 1995 Lion-Francois et al., 2010 Fleischhauer et al., 1998	94.5%
	Calmodulin	**(1471–1523)** **DNF—IFD** **(53 aa)** Potet et al., 2009	L1501V, I1521K G1502S DQKP 1507-1509 R1512W F1520L	BrS BrS LQT3 BrS DCM	Kapplinger et al., 2010 Smits et al., 2005 Keller et al., 2003 Deschênes et al., 2000 McNair et al., 2011	Equivalent sequence: (1296–1348) DNF—VYD (53 aa)	G1306V T1313M T1313A	PC PC PC	Plassart et al., 1994 Fukudome et al., 2003 Bouhours et al., 2004	94.5%
DIV S5-S6 loop	Navβ1	Equivalent sequence: (1720–1748) ILN—AVG (29 aa)	G1712S	BrS	Kapplinger et al., 2010	**(1545–1574)** **ILN—SIG** **(30 aa)** Makita et al., 1996				90.1%
C-ter	FGF12/13	**(1784–1864)** **EPL—LGE** **(81 aa)** Liu et al., 2003 Wang et al., 2011	E1784K E1784K E1784K S1787N D1790G 1795insD 1795insD Y1795C L1825P R1826H Q1832E, V1861I D1840G	BrS LQT3 L/B LQT3 LQT3 L/B LQT3 L/B LQT3 LQT3 BrS LQT3	Priori et al., 2002 Splawski et al., 2000 Makita et al., 2008 Splawski et al., 2000 An et al., 1998 Bezzina et al., 1999 van Langen et al., 2003 Rivolta et al., 2001 Makita et al., 2002 Ackerman et al., 2001 Kapplinger et al., 2010 Benhorin et al., 1998	Equivalent sequence: (1610–1690) EPL—LGD (81 aa)				95.1%
C-ter	Calmodulin	**(1908–1919)** **IQ-motif IQRAFRRHLLQR** **(12aa)** Tan et al., 2002 Young and Caldwell, 2005	Q1909R R1913H	LQT3 LQT3	Tester et al., 2005 Napolitano et al., 2005	**(1734–1745)** **IQRAYRRHLLQR** **(12aa)** Young and Caldwell, 2005				100.0%
	Nedd4-2	**(1974-1980)** **PY-motif** **PPSYDSV** **(7 aa)** van Bemmelen et al., 2004				No homology				No homology
	Syntrophin	**(2014–2016)** **SIV** **(3 aa)** Ou et al., 2003				**(1834–1836)** **SLV** **(3 aa)** Gee et al., 1998				100.0%
	PTPH1	**(2014–2016)** **SIV** **(3 aa)** Jespersen et al., 2006				Equivalent sequence: (1834–1836) SLV (3 aa)				100.0%
	SAP97	**(2014–2016)** **SIV** **(3 aa)** Petitprez et al., 2011				Equivalent sequence: (1834–1836) SLV (3 aa)				100.0%

For each channel the identified binding site **(in bold)** and the equivalent sequence on the channel counterpart are presented. The % amino acid sequence similarities between Nav1.5 (NCBI Reference Sequence NP_932173.1) and Nav1.4 (NP_000325.4) channels were estimated using the following website: http://www.ch.embnet.org/software/LALIGN_form.html. Nav1.4 interaction with ankyrin is only suggested by chimeric constructs, it remains to be studied with full length proteins (Lemaillet et al., 2003). It is noticeable that Nedd4-2 consensus binding site “PPSYD(E in Nav1.8)S(R in Nav1.1)” is present in all human Nav channels except Nav1.4. Single amino acid mutations identified in human disease in each binding site are reported. DI to DIV, domains I to IV; DI(S5-S6) and DIV(S5-S6), extracellular connecting loops between S5 and S6 intramembrane segments in domains I and IV; ID, intracellular interdomains; N-ter and C-ter, N- and C-terminus ends; BrS, Brugada Syndrome; AF, Atrial Fibrillation; LQT3, Type 3 long QT syndrome; L/B, Overlap of LQT3 and BrS; DCM, Dilated Cardiomyopathy; SCM, Sodium Channel Myotonia; SNEL, Sporadic Neonatal Episodic Laryngospasm; PC, Paramyotonia Congenita; SNDM, Severe neonatal Non-Dystrophic Myotonia.

## Comparison of missense mutations. are there (dys-) functional homologies between Nav1.4 and Nav1.5?

Around 300 mutations in *SCN5A* have been identified in patients presenting with BrS, 31% being frameshift, nonsense or splice-site mutations, and 69% being missense or rarely in-frame deletions/insertions (Kapplinger et al., 2010). When studied in patch-clamp in heterologous expression systems, mutated Nav1.5 channels are showing different types of loss of function, such as a decrease in current density, a positive shift in the activation curve, a negative shift in the inactivation curve, or a loss of regulation by PKA (Tarradas et al., 2013; Zeng et al., 2013; Aiba et al., 2014). Mutations in *SCN5A* have also been found in patients presenting with LQT syndrome (named LQTS3 when *SCN5A* is mutated). As opposed to BrS, mutated channels in LQTS3 patients show a gain a function, mainly through an increase in a persistent Na^+^ current (cf. Part Clinical Description of the Main Nav1.4 and Nav1.5 Related Pathologies). As a result, **BrS** mutations are associated to membrane **hypo-excitability**, whereas **LQTS3** mutations are associated to prolonged action potential, referred here as membrane **hyper-activity**.

In the skeletal muscle, a similar binary classification is observable among the 70 mutations identified so far, that are nearly exclusively missense or rarely in-frame deletions/insertions. **NDM** are linked to membrane **hyper-excitability**, often due to defective inactivation and hence **a gain of function** of Nav1.4 channel activity (Clarke et al., 2011). On the contrary, **hypoPP** is linked to membrane **hypo-excitability**, and is often due to the apparition of an aberrant current through the gating pore that can be a proton or a monovalent cation current (Sokolov et al., 2007). This so-called “omega” current (or gating pore current) causes paradoxical depolarization of myofibers in low K^+^, which inactivates Nav1.4 and renders myofibers non excitable. Seemingly paradoxical, **hyperPP** is associated with **gain of function of Nav1.4** (as observed for myotonia) but loss of function on skeletal muscles (paralysis). As for myotonia, defective inactivation of Nav1.4 is often observed and favors membrane depolarization. The paradox is resolved if we consider that wild type Nav1.4 channels will be more inactivated due to a slightly more depolarized membrane, thus causing a loss of sarcolemmal excitability and myofiber paralysis (Cannon, 2015). The development of myotonia or hyperPP may depend on the degree of membrane excitability. This has been suggested for instance when in the same family, females carrying the M1370V mutation develop only a myotonia (PC) whereas males are presenting with both myotonia and hyperPP (Okuda et al., 2001).

**Figure 2:**
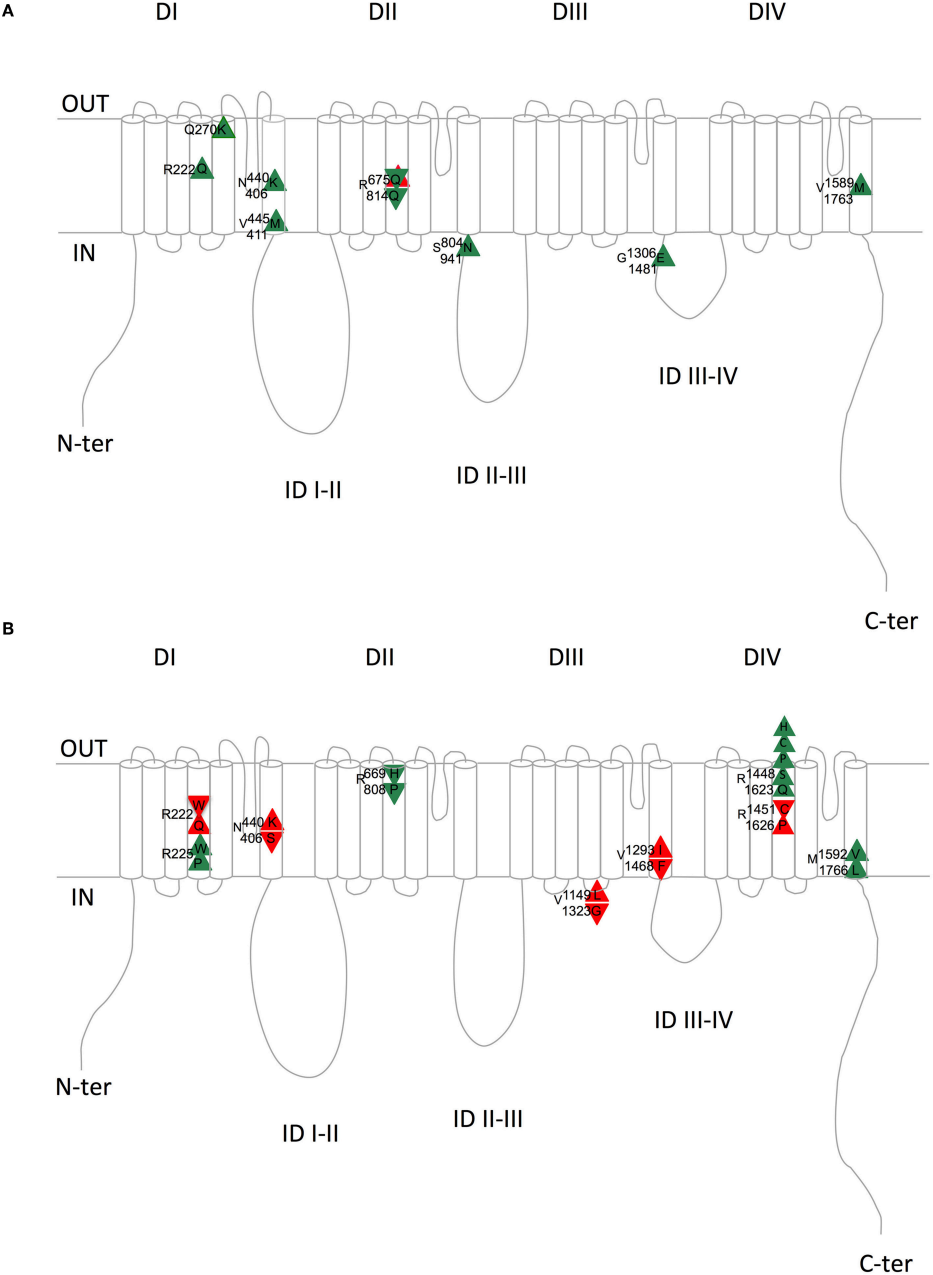
**Schematic representation of the equivalent Nav1.4/Nav1.5 amino acids with similar (A) or divergent (B) mutations in patients with skeletal (Nav1.4) or cardiac (Nav1.5) pathologies**. Upward triangles indicate a gain of function on membrane excitability and downward triangles a loss of function on membrane excitability. Green and red triangles indicate consistent and inconsistent effects on Nav1.4 and Nav1.5 regarding membrane excitability, respectively. Upper amino acid number/letter, Nav1.4; Lower amino acid number/letter, Nav1.5. DI to DIV, domains I to IV; ID, intracellular interdomains; N-ter and C-ter, N-and C-terminus ends.

Nav1.4 and Nav1.5 are similar. If we consider the aligned region between Nav1.5 and Nav1.4, which represents 95% of Nav1.4 sequence, 67% of the amino-acids are identical. Knowing that, one can wonder whether mutations have been identified at equivalent positions in both channels, and whether, in this case, the new amino-acid is the same, such as Q270K in both Nav1.4 and Nav1.5 or V445M in Nav1.4 and V411M in Nav1.5 (V445 is aligned with V411). It is possible to use an online compilation that has been proposed using a paralog annotation approach in order to retrieve homologous or nearly homologous variants in both genes (Ware et al., 2012; Walsh et al., 2014). If the same mutations of homologous residues exist, do they give rise to similar dysfunction on both channels? If yes, we can expect that both mutations give rise to the same change in membrane excitability. For instance, if a Nav1.5 mutation leads to hyper-activity of cardiac cells (LQTS3), the corresponding mutation in Nav1.4 may also give rise to a hyper-excitability phenotype of the skeletal muscle cells such as HyperPP, PC, or SCM. Tables 4, 5 present **all the corresponding amino acids found to be mutated** in patients with cardiac (Nav1.5) or neuromuscular (Nav1.4) pathologies. Table 4 and Figure 2A list the mutations for which the amino acid substitutions are the same, and Table 5 and Figure 2B those for which they are divergent.

**Table 4:** **List of equivalent amino acids found to be similarly mutated in patients with cardiac (Nav1.5) or skeletal (Nav1.4) pathologies**.

**Region**	**Nav1.4**	**Pathology**	**References**	**Nav1.5**	**Pathology**	**References**
Domain I S4	R222Q	Myotonia	Durran et al., 2011	R222Q	MEPPC	Laurent et al., 2012
Domain I S5	Q270K	PC	Carle et al., 2009	Q270K	LQT3	Kapplinger et al., 2010; Calloe et al., 2011
Domain I S6	N440K	Normo Hyper PP	Lehmann-Horn et al., 2011; Lossin et al., 2012	N406K	LQT3	Kato et al., 2014
Domain I S6	V445M	SCM	Takahashi and Cannon, 1999	V411M	LQT3	Horne et al., 2011
Domain II S4	R675Q	Normo, Hyper or Hypo PP?	Vicart et al., 2004; Wu et al., 2014	R814Q	BrS/ CM-AF	Frigo et al., 2007
Interdomain II-III	S804N	SCM	Fournier et al., 2006	S941N	LQT3/de novo SIDS	Schwartz et al., 2000
Interdomain III-IV	G1306E	SCM SNEL	Mitrovic et al., 1995; Fleischhauer et al., 1998	G1481E	LQT3	Kapplinger et al., 2009
Domain IV S6	V1589M	Overlap PC-SCM	Heine et al., 1993; Mitrovic et al., 1994; Hayward et al., 1999	V1763M	LQT3	Chang et al., 2004; Ma et al., 2013

Same amino acid substitutions occurring in both channels lead to consistent pathologies (in green) regarding membrane excitability. PC, Paramyotonia Congenita; MEPPC, Multifocal Ectopic Purkinje-related Premature Contraction; LQT3, Type 3 Long QT syndrome; SIDS, Sudden Infant death syndrome; Hypo, Normo, Hyper PP, Hypo, Normo, Hyper-kalemic Periodic Paralysis; SCM, Sodium channel Myotonia; BrS, Brugada Syndrome; CM, Cardiomyopathy; AF, Atrial Fibrillation; SNEL, Sporadic Neonatal Episodic Laryngospasm.

**Table 5:** **List of equivalent amino acids found to be differently mutated in patients with cardiac (for Nav1.5) or neuromuscular (for Nav1.4) pathologies**.

**Region**	**Nav1.4 mutation**	**Pathology**	**References**	**Nav1.5 mutation**	**Pathology**	**References**
IS4	R222W	Hypo PP	Matthews et al., 2009	R222Q	MEPPC	Laurent et al., 2012
IS4	R225W	SCM	Lee et al., 2009	R225P	LQT3	Beckermann et al., 2014
IS6	N440K	Normo Hyper PP	Lehmann-Horn et al., 2011; Lossin et al., 2012	N406S	BrS	Itoh et al., 2005b
IIS4	R669H	Hypo PP	Struyk et al., 2000; Kuzmenkin et al., 2002	R808P	BrS	Kapplinger et al., 2010
III inter S4-S5	V1149L	HyperPP with myotonia	Yoshinaga et al., 2015	V1323G	BrS	Kapplinger et al., 2010
IIIS6	V1293I	SCM	Koch et al., 1995; Green et al., 1998	V1468F	BrS	Kapplinger et al., 2010
IVS4	R1448H	PC	Ptácek et al., 1992; Chahine et al., 1994; Mohammadi et al., 2003; Holzherr et al., 2014	R1623Q	LQT3	Kambouris et al., 1998; Makita et al., 1998
IVS4	R1448C	PC	Ptácek et al., 1992; Chahine et al., 1994; Featherstone et al., 1998	R1623Q	LQT3	Kambouris et al., 1998; Makita et al., 1998
IVS4	R1448P	PC	Featherstone et al., 1998	R1623Q	LQT3	Kambouris et al., 1998; Makita et al., 1998
IVS4	R1448S	PC (mild)	Bendahhou et al., 1999	R1623Q	LQT3	Kambouris et al., 1998; Makita et al., 1998
IVS4	R1451C	Hypo PP	Arzel-Hézode et al., 2009	R1626P	LQT3	Ruan et al., 2007
IVS6	M1592V	Normo Hyper PP	Rojas et al., 1991; Cannon and Strittmatter, 1993; Hayward et al., 1999; Rojas et al., 1999	M1766L	LQT3	Valdivia et al., 2002; Ye et al., 2003

Divergent amino-acid substitutions occurring in the two channels lead either to consistent (in green) or inconsistent (in red) pathologies regarding membrane excitability. Hypo, Normo, Hyper PP, Hypo, Normo, Hyper-kalemic Periodic Paralysis; MEPPC, Multifocal Ectopic Purkinje-related Premature Contraction; SCM, Sodium channel Myotonia; PC, Paramyotonia Congenita; LQT3, Type 3 Long QT syndrome; BrS, Brugada Syndrome.

When looking at Table 4 and Figure 2A, it is striking to observe that all paralog mutations give rise to clearly consistent functional effects, except one which is at first unclear, as detailed below. Indeed, all Nav1.4 mutations linked to membrane hyper-excitability (PC, SCM, and HyperPP) correspond to Nav1.5 mutations linked to membrane hyper-activity (LQTS3), except R675Q (R814Q in Nav1.5). The comparison between Nav1.4 R675Q and Nav1.5 R814Q is not obvious because the pathology induced by Nav1.4 R675Q mutation is difficult to classify as Normo/Hyper PP or Hypo PP (Vicart et al., 2004). Indeed, patients experienced normal as well as decreased potassium levels concomitant to attacks. The rat ortholog of human Nav1.4 R675Q generates an omega current activated by depolarization when expressed in *Xenopus* oocytes (Sokolov et al., 2008) (cf. above). The omega current represents less than 1% of the peak pore current but it remains constant after slow inactivation of the pore current and requires high hyperpolarizations to deactivate. Therefore, it is suspected that this current, carried by Na^+^ and K^+^ ions, maintained during trains of action potentials and with a residual non-deactivated activity at resting potential could lead to sodium accumulation and a decrease in membrane excitability. It will be interesting to test whether the corresponding mutation in Nav1.5 is also responsible for an omega current. Moreover, the R675Q Nav1.4 mutation gives rise to a hyperpolarizing shift of the inactivation curve and a slower recovery from inactivation when expressed in HEK293 cells. (Vicart et al., 2004; Wu et al., 2014). Altogether, these observations suggested us to rank it as a hypo-excitability causing mutation, consistent with the BrS phenotype (loss of function) induced by the homologous Nav1.5 mutation R814Q.

Table 4 and Figure 2A summarize the (dys-)functional homology between the equivalent mutant in Nav1.4 and Nav1.5. On the contrary, Table 5 and Figure 2B show that divergent amino acid substitution at the equivalent position leads to some inconsistencies (in red, 5/12). This suggests that the nature of the amino acid substitution is determinant for the direction of the functional net effect (loss or gain of function).

At last, we focused on five equivalent mutations that have been studied extensively in patch-clamp in both Nav1.4 and Nav1.5. Table 6 shows changes in each biophysical parameter for these mutations. When looking at the direction of the functional effects (gain or loss of function), we observe two major points. First, a strikingly similar functional effect of the same mutations in both channels. Second, all the gain of function mutations leading to hyper-activity/excitability provoke an increase in the persistent current, when measured, suggesting that this mechanism plays a major role in the pathogenesis of Nav channelopathies.

**Table 6:** **Variations of biophysical parameters compared to wild type channels for five equivalent mutations identified in Nav1.4 and Nav1.5 that have been studied extensively in patch clamp**.

**Subunit**	**Mutation**	**Pathologies**	**Cell model**	**ΔV0.5 act (mV)**	**ΔV0.5 fast inac (mV)**	Mutant/WT Fast inact tau ^*^	**Mutant/WT i persistent**	**References**
Nav1.4	I141V	SCM	HEK	−10	0	77% at −10 mV	?	Petitprez et al., 2008; Amarouch et al., 2014
Nav1.5	I141V	ExPVC	HEK	−8	0	86%	?	Amarouch et al., 2014; Swan et al., 2014
Nav1.4	Q270K	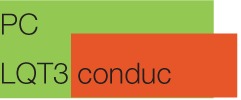	HEK	1.3	12.5	168% at −25 mV	200%	Carle et al., 2009
Nav1.5	Q270K		CHO	5.8	9.9	260% at −25 mV	338%	Calloe et al., 2011
Nav1.4	N440K	Normo Hyper PP	HEK	0	7.1	100%	800%	Lossin et al., 2012
Nav1.5	N406K	LQT3	CHO	8.6	0	217%	550%	Kato et al., 2014
Nav1.4	V445M	Myotonia	HEK	−4.1	−4.9	?	1400%	Takahashi and Cannon, 1999
Nav1.5	V411M	LQT3	HEK	−8.1	−7.9	75%	176%	Horne et al., 2011
Nav1.4	V1589M	overlap PC-SCM	HEK	0	5.4	100%	362%	Mitrovic et al., 1994
Nav1.5	V1763M	LQT3	hiPSC-CMs	0	16.8	?	486%	Ma et al., 2013

Recording were all done at room temperature (except for Nav1.4 N440K: not indicated).

* Fast inactivation tau is measured at −30 mV except when indicated.

Green, consistent effect; red, inconsistent effect; SCM, Sodium Channel Myotonia; ExPVC, exercise-induced polymorphic ventricular premature complexes; PC, Paramyotonia Congenita; LQT3, Type 3 Long QT syndrome; Normo, Hyper PP, Normo, Hyper-kalemic Periodic Paralysis; Conduc, conduction disease; hiPSC-CMs, cardiomyocytes generated from human induced pluripotent cells; ?, means not determined.

Recently, an omega current has been observed in Nav1.5 mutant channels identified in patients presenting with arrhythmic DCM or MEPPC (Gosselin-Badaroudine et al., 2012b; Moreau et al., 2015b). This omega current, due to mutations of arginine in the S4 of domain I, is similar to the one observed in Nav1.4 (Sokolov et al., 2007; Struyk et al., 2008; Francis et al., 2011; Gosselin-Badaroudine et al., 2012a; Groome et al., 2014). This further strengthens the functional similarity between Nav1.4 and Nav1.5 in pathophysiological situations. A common feature of MEPPC, is an increase in window current provoked by the Nav1.5 R225W, R222Q, and R225P mutations, increasing cardiac excitability of the fascicular-Purkinje system (Laurent et al., 2012; Mann et al., 2012). Another common feature of two of these mutations: R222Q and R225W is the presence of an omega current. This Nav1.5 omega current may be responsible for the peculiar cardiac phenotype (Moreau et al., 2015a), similar to the omega current of Nav1.4 being responsible for the hypoPP phenotype, through sodium accumulation and a decrease in membrane excitability (Sokolov et al., 2008). Indeed, most of the *SCN5A* mutations linked to DCM are located in the voltage sensor domain (VSD) as pointed by McNair et al. (2011). However, in some cases DCM may be secondary to arrhythmias and window current increase. For instance, preventing arrhythmias by quinidine improved the ventricular function (ejection fraction) in patients with the Nav1.5 R222Q mutation, *via* a decrease in the window sodium current (Laurent et al., 2012). The use of specific inhibitor of the alpha pore and the omega (or gating pore) current would allow to test for the respective role of the altered gating (activation, inactivation) and the omega current on the development of the pathology. Noteworthy, the various localization of the Nav1.4 mutations giving rise to omega current (in domains I, II, and III) strongly suggests that similar mutations in Nav1.5 will be identified in domain II and III in addition to the ones already identified in domain I (Moreau et al., 2015b).

To conclude, given the sequence similarity between Nav1.4 and Nav1.5, any characteristics described for one channel subunit may shed light on the properties of the counterpart channel subunit, such as the presence of specific protein partners, or the effects of a specific amino acid substitution. One can argue that the effect of a mutation on Nav1.4 is difficult to compare with Nav1.5 since the different molecular and cellular environment may drastically modify the effect of the mutation. Nevertheless, we noticed that the same mutation lead to comparable effect regarding membrane hypo or hyper-excitability (Table 4 and Figure 2A). This suggests that the cellular environment is usually not able to invert the effect of a mutation from gain to loss of function phenotypes and reciprocally. Such comparison between Nav1.4 and Nav1.5 will probably draw more and more interest, to address the challenge of interpreting and understanding pathogenicity of rare *SCN4A* or *SCN5A* variants revealed by next-generation sequencing studies (Arnold et al., 2015; Bergareche et al., 2015; Coll et al., 2015).

## Author contributions

Parts were written by: Part I : DS, YP, VF (Nav1.4), FC (Nav1.5). Part II: SN (Nav1.4), GT (Nav1.5). Part III: FL, CM. Part IV: DS (Nav1.4), JB, OM, IB, GL (Nav1.5). DS and GL initiated the project. IB, FC, JB, YP critically read the entire Manuscript. GL supervised the Ms.

## Funding

This work was supported by INSERM, CNRS, the Fondation d'entreprise Génavie, the Fondation pour la Recherche Médicale (PLP20141031304), the Association Française contre les Myopathies - Téléthon (16495), the 7th European Community Framework Programme (PIOF-GA-2011-298280, PIRG06-GA-2009-256397, HEALTH-F2-2009-241526), the ANR (ANR-12-BSV1-0013-01), Investissements d'avenir (ANR-10-IAIHU-06), and Nantes and Sorbonne universities, UPMC-Paris 06.

### Conflict of interest statement

The authors declare that the research was conducted in the absence of any commercial or financial relationships that could be construed as a potential conflict of interest.
